# Biophilia as a temperament trait, not a universal: children’s attraction and aversion to biodiversity in the United States and Democratic Republic of Congo

**DOI:** 10.3389/fpsyg.2026.1904601

**Published:** 2026-10-30

**Authors:** Vanessa Woods, Brian Hare, Gráinne M. Fitzsimons, Melinda J. Knuth

**Affiliations:** 1 Department of Evolutionary Anthropology, Duke University, Durham, NC, United States; 2 Department of Psychology and Neuroscience, Duke University, Durham, NC, United States; 3 Fuqua School of Business, Duke University, Durham, NC, United States; 4 Department of Horticultural Science, North Carolina State University, Raleigh, NC, United States

**Keywords:** biodiversity, biophilia, child development, conservation psychology, cross-cultural research, environmental psychology

## Abstract

**Introduction:**

The Biophilia Hypothesis proposes that humans have a universal attraction to the natural world, yet little is known about how this attraction emerges or varies during childhood. We tested the Biophilia Reactivity Hypothesis, which reframes biophilia as an early-emerging, individually variable, temperament-like orientation toward biodiversity.

**Methods:**

We studied 295 children aged 3-13 years in two distinct settings: 124 children at the North Carolina Zoo in the United States and 171 children at Lola ya Bonobo Sanctuary in Kinshasa, Democratic Republic of Congo. Children completed a biophobia measure adapted from the Revised Fear Survey Schedule and a novel image-based biophilia task assessing Backyard Biophilia, Wildlife Tolerance, and Biophilic Travel.

**Results:**

Children showed substantial individual variation across biophilia and biophobia measures, with responses spanning the available scale ranges. U.S. children showed higher Wildlife Tolerance and Biophilic Travel scores than Congolese children, whereas Backyard Biophilia did not differ between groups. Patterns of animal fear also differed between samples: U.S. children reported greater fear of some insects and arthropods, whereas Congolese children reported greater fear of several predators and reptiles. Biophobia was not significantly correlated with any of the three biophilia domains in either sample, indicating that low fear does not necessarily imply greater attraction to biodiversity.

**Discussion:**

These findings support a model of biophilia as an individually variable, multidimensional disposition rather than a uniform human preference. They also suggest that attraction to biodiversity and fear of animals are partly distinct psychological systems and highlight the importance of studying children’s biodiversity preferences across development in varying ecological and sociocultural contexts.

## Introduction

Biodiversity loss is a critical threat to our future (World Economic Forum, 2022). It reduces carbon storage, increases vulnerability to environmental stressors, and has adverse effects on human health (Cardinale et al., 2012; Weiskopf et al., 2024; Frank, 2024; Tilman et al., 2014). Despite numerous national and international treaties, there has been little progress in slowing species decline (Gillespie, 2013; Secretariat of the Convention on Biological Diversity, 2020). Biodiversity conservation requires large-scale cooperation involving individuals, communities, and countries. Researchers have called for a deeper understanding of human psychology as it relates to biodiversity conservation (Saunders, 2003), but there is still a lack of research explicitly exploring this relationship (Selinske et al., 2018).

The most significant attempt to do so is the Biophilia Hypothesis. First proposed by E. O. Wilson in 1984, the Biophilia Hypothesis was framed as a conservation ethic, the “innately emotional affiliation of human beings to other living organisms” (Wilson, 2007). According to Wilson, biophilia is ‘a universal instinct shared by all’ (Wilson, 1984). The Biophilia Hypothesis as a categorical human universal has inspired extensive research on the psychological benefits of nature exposure (Russell et al., 2013; Capaldi et al., 2015; Reyes-Riveros et al., 2021). However, empirical support for its prediction remains limited (Joye and De Block, 2011), in part due to the lack of a consistent definition of “nature,” negative results, and cross-cultural research (Wendelboe-Nelson et al., 2019; Gascon et al., 2015; Clark et al., 2007).

Environmental psychology is the exploration of the relationship between people and their environment, which includes both built and natural environments (Stokols, 1978), and as an interdisciplinary field, is uniquely placed to examine biophilia. Research on natural environments has mostly focused on the benefits of nature, such as attention restoration (Kaplan, 1995) and stress recovery (Ulrich et al., 1991) [although negative effects of nature have also been explored (Bixler and Floyd, 1997)]. Research in environmental psychology occasionally references the Biophilia Hypothesis as an evolutionary explanation for the human affinity for the natural world (Ulrich, 1993). Increasingly, biophilia is referenced in areas of environmental psychology related to architecture and design (Kellert and Calabrese, 2015). However, research on biophilia has generally retained the original framing of biophilia as a human universal, with less attention to individual variability.

We propose the Biophilia Reactivity Hypothesis (BRH) as an adjustment to the original hypothesis (Woods and Knuth, 2023). The BRH redefines biophilia as a temperament trait: a biologically and environmentally influenced attraction toward biodiversity. This reframing addresses observed discrepancies in pro-nature attitudes and conservation behavior (Colombo et al., 2023; Nisbet et al., 2009). Not everyone is equally drawn to the natural world; some people are indifferent or even biophobic (Barrable and Booth, 2022). The BRH conceptualizes biophilia as a continuum, similar to traits such as novelty seeking or sociability, allowing for both affinity and aversion to biodiversity (Table 1).

**Table 1:** Biophilia reactivity spectrum: from attraction to aversion.

Measure	Biodiversity attraction	Biodiversity aversion
Biophobia	No fear of animals, including those commonly associated with specific phobias (e.g., snakes, spiders).	Slight or serious fear toward one or more animals (including insects and arthropods).
Biophilia factors		
Wildlife Tolerance	Willingness to welcome a wide range of animals into the local environment, including potentially dangerous species (e.g., snakes, wolves, elephants).	Preference for familiar, non-threatening animals and avoidance of species perceived as dangerous (e.g., butterflies, deer, okapi).
Backyard Biophilia	Interest in cultivating gardens that support high biodiversity and attract various animal species (e.g., pollinator gardens, woodlands).	A preference for gardens with low biodiversity that are unlikely to support animal diversity (eg, lawns)
Biophilic Travel	A desire to visit the kind of environments most likely to house the kind of biodiversity they are attracted to (eg. rainforests, coral reefs)	A preference for built environments, such as cities.

This table illustrates the proposed continuum of biophilic reactivity, ranging from attraction to biodiversity to aversion. It highlights variability across domains, including fear of animals (biophobia), a tolerance for wildlife in and around the home environment (Wildlife Tolerance), preferences for biodiversity in gardens (Backyard Biophilia), and travel motivations (Biophilic Travel).

According to Goldsmith et al. (1987), “Temperament consists of relatively consistent, basic dispositions inherent in the person that underlie and modulate the expression of activity, reactivity, emotionality, and sociability.” The criteria for a temperament trait are: (1) individual variability, (2) early emergence, (3) stability over time, and ideally, (4) physiological signature of neurobiological mechanisms. This study focuses on the first two.

To understand the opposite end of the biophilia spectrum, we examine biophobia, defined as fear of biodiversity. Research related to biophilia rarely considers biophobia, even though animal fears, including fears of insects and arthropods, are among the most common childhood fears, emerge early in life (Ollendick et al., 2002; Muris et al., 2002), and are distinct from other fears (situational, environmental) (Ollendick et al., 2013), perhaps with different neural pathways than non-animal fears (Lueken et al., 2011). Using the Revised Fear Survey Schedule (FSSC-R), researchers have found that fear patterns vary cross-culturally, influenced by experience, culture, and local ecologies (Shore and Rapport, 1998; Burnham et al., 2016).

The Biophilia Reactivity Hypothesis predicts that biophilia and biophobia may be at opposite ends of a continuum—meaning lower fear should correlate with stronger attraction to biodiversity. However, research on animal domestication challenges this logic: the process of domestication requires more than an absence of fear. It requires that attraction replaces fear (Hare, 2017). In humans, too, attraction to biodiversity may require more than just the absence of fear. To go beyond measuring how afraid or unafraid an individual is toward biodiversity, we must actively measure *attraction* to biodiversity. Therefore, we developed a second instrument to capture this dimension.

While studies on children’s relationship to nature are increasing, few explicitly examine attraction to biodiversity. Most are conducted in developed countries and tend to emphasize group-level averages rather than exploring individual variability (Kahn and Kellert, 2002; Tillmann et al., 2018). Findings are mixed: some suggest children prefer familiar gardens over more biodiverse settings (Hand et al., 2017), while others report a stronger interest in exotic species, which may enhance conservation motivation (Ballouard et al., 2011). Still others find that children are drawn to ecological novelty and variety (Beery and Jørgensen, 2018). Oña et al. (2024) review these inconsistencies and highlight persistent gaps in the developmental literature, including the lack of cross-cultural studies, limited inclusion of young children, and a tendency to overlook individual differences in favor of group-level trends. Our study directly addresses these gaps by testing the Biophilia Reactivity Hypothesis using a novel, image-based instrument designed to measure early-emerging, domain-specific biophilic preferences in children across two ecologically and culturally distinct populations.

To capture this variability, we developed a three-part biophilia instrument measuring: (1) Backyard Biophilia (interest in biodiversity in domestic gardens), (2) Wildlife Tolerance (tolerance and attraction to wildlife near one’s home), and (3) Biophilic Travel (interest in visiting biodiverse destinations).

The Democratic Republic of Congo (DRC) provides an ideal test case. It harbors the highest biodiversity in Africa and the world’s second-largest rainforest but is also experiencing rapid urbanization and deforestation (De Wasseige et al., 2014; Tchatchou et al., 2015). Kinshasa, the capital, is a rapidly growing megacity with declining tree cover (Global Forest Watch, 2023). Despite its ecological significance, no prior studies have assessed children’s psychological orientation to biodiversity in this region.

## Hypotheses and predictions

The Biophilia Reactivity Hypothesis proposes that biophilia is a temperament trait marked by early-emerging individual differences in attraction to biodiversity. Accordingly, this study takes a deductive, hypothesis-driven approach, testing predictions derived from the BRH. Using two instruments, this study tests the following predictions:

### Individual variability

If biophilia is a temperament trait, it should show meaningful variation across individuals. Like other traits such as sociability or fearfulness, we expect scores to be normally distributed. An absence of ceiling or floor effects would suggest the instruments are capturing this full range.

- Biophilia and biophobia scores will follow normal distributions.
- No ceiling or floor effects will be observed.

### Early emergence

The age range of 3–13 was selected to capture the emergence and expression of early temperament traits. Preschool age is a common starting point for developmental studies, as children can use language to express preferences, participate in structured tasks, and show observable stability in traits like sociability, novelty seeking, and emotional reactivity (Goldsmith et al., 1987; Derryberry and Rothbart, 1988). Extending through late childhood allows us to examine how these traits manifest across developmental stages, while avoiding the confounding influences of adolescence.

- Individual differences will be evident in children aged 3 to 13.

### Cross-cultural differences

The Biophilia Reactivity Hypothesis suggests that living in a biodiversity-rich region does not necessarily translate into stronger biophilia (Woods and Knuth, 2023). In urban environments like Kinshasa, biodiversity may be perceived as threatening, irrelevant, or even absent from daily life. Thus, we predicted cross-cultural differences in biophilia and biophobia but did not assume that children in high-biodiversity contexts would necessarily exhibit stronger biophilic responses.

- Biophilia and biophobia scores will differ between children in the U. S. and the DRC.
- Biophilia measures will be more sensitive to cultural and contextual variation than biophobia measures, which may reflect more universal aversions.

### Biophilia factor correlations

If biophilia reflects an underlying temperament trait, we expect consistent preferences across different but related domains of biodiversity. Children who are drawn to biodiversity in familiar settings (e.g., backyards) may also show interest in wildlife encounters and biodiverse travel destinations. Thus, scores on Backyard Biophilia, Wildlife Tolerance, and Biophilic Travel are predicted to be positively correlated, reflecting a coherent, trait-like disposition toward biodiversity.

- Scores on Backyard Biophilia, Wildlife Tolerance, and Biophilic Travel will be positively correlated.

### Biophilia vs. Biophobia

The Biophilia Reactivity Hypothesis predicts that biophilia and biophobia lie at opposite ends of a single continuum—meaning that children who are less fearful of animals should also be more strongly attracted to biodiversity, and vice versa. This assumption aligns with the idea that reduced fear facilitates engagement with the natural world.

- Biophilia should have a moderate to strong inverse correlation with biophobia

## Methods

### Ethics statement

This study was approved by the Duke University Institutional Review Board (IRB Protocol #2018-0035, *Dehumanization among children*). All procedures involving human participants were conducted in accordance with the ethical standards of the institutional research committee and the 1964 Helsinki Declaration and its later amendments. Written informed consent was obtained from a parent or legal guardian for participants in the United States, and from schoolteachers acting in loco parentis for participants in the Democratic Republic of Congo. Verbal assent was obtained from all children prior to participation.

### Participants

We tested 310 children aged 3 to 13 years, with 295 included in the final sample after excluding incomplete responses and age outliers. U. S. participants (n = 124) were recruited from the North Carolina Zoo, (Asheboro, NC, USA); DRC participants (n = 171) were recruited during school-organized field trips to the Lola ya Bonobo Sanctuary (Kinshasa, DRC). Concise demographics are presented in Table 2. Full demographic information, including excluded cases, is available in Supplementary Table A3. Participants were recruited opportunistically from visitors to the North Carolina Zoo and from school-organized visits to the Lola ya Bonobo Sanctuary. Sample size was determined by practical and logistical constraints at the field sites rather than an *a priori* power analysis, which we acknowledge as a limitation.

**Table 2:** Participant demographics by country.

Characteristic	Total		US		Congo	
	(*n* = 295)		(*n* = 124)		(*n* = 171)	
	*n*	%	*n*	%	*n*	%
Gender						
Male	139	47%	57	46%	82	48%
Female	156	53%	67	54%	89	52%
Age, years						
3	12	4%	12	10%	0	0%
4	11	4%	11	9%	0	0%
5	16	5%	15	12%	1	1%
6	20	7%	20	16%	0	0%
7	11	4%	9	7%	2	1%
8	21	7%	16	13%	5	3%
9	25	8%	14	11%	11	6%
10	61	21%	14	11%	47	27%
11	51	17%	3	2%	48	28%
12	51	17%	7	6%	44	26%
13	16	5%	3	2%	13	8%

This table summarizes demographic characteristics of the final study sample (*N* = 295), including gender and age distribution across participants from the United States (*n* = 124) and the Democratic Republic of Congo (*n* = 171).

### Field sites

The North Carolina Zoo spans 500 forested acres and emphasizes conservation education. Children in this sample were visitors to the zoo accompanied by their parents, making it likely that they already held some interest in animals and nature. Given the zoo’s immersive, conservation-oriented setting and its appeal to families with an interest in wildlife, children recruited from this site might be more positively predisposed toward biodiversity and wildlife-related experiences than the general population. Also, zoo admission requires a paid ticket, so participation also implied a minimum level of socioeconomic resources and a willingness to pay for access to wildlife. Children were recruited onsite, and parental consent was obtained prior to participation. Testing occurred in a quiet room near the chimpanzee exhibit.

Lola ya Bonobo is a 75-acre sanctuary for orphaned bonobos near Kinshasa. The children in this sample were students from local schools whose entire class visited the sanctuary as part of a school-organized field trip. The sanctuary covered all admission and transportation costs, so children were not selected based on their families’ ability or willingness to pay. This sample likely represented a broader range of socioeconomic backgrounds, with less self-selection based on interest or investment in wildlife experiences.

Testing occurred in an open-air shelter near the sanctuary entrance. Before each school visit, educators contacted schools to determine student interest. Children were brought individually to the shelter for testing. Teachers provided signed consent prior to the visit. The study was conducted in French, the national language of the DRC, by the researcher, with the exception of two schools from low-income, underprivileged communities (*n* = 64) who spoke only Lingala. In these cases, an educator (who spoke both French and Lingala) served as the interpreter and was trained by the researcher to conduct the experiment.

In both the US and Congo sample, any spontaneous comments made by children during the testing session were informally noted by the experimenter.

### Instrument 1: biophobia

To test children’s fear of biodiversity, we used a subset of the Revised Fear Survey Schedule (FSSC-R) (Ollendick, 1983). The FSSC-R is an 80-item survey instrument designed to assess the number and intensity of fears in children and adolescents. In this study, responses were scored on a three-point scale: 0 = Not afraid, 1 = Afraid, and 2 = Very afraid. Of the 80 items, 9 items related to animals; lizards, snakes, bears or wolves, spiders, bats or birds, cats, ants or beetles, worms or snails, rats or mice. For the purpose of this study, items were extracted from the survey that related to specific animal fears and phobias (Ollendick, 1983). For this study, we excluded the ‘worms or snails’ and ‘rats or mice’ items and treated the three paired items (‘bears or wolves,’ ‘bats or birds,’ and ‘ants or beetles’) as separate animal categories. This yielded 10 categories: lizards, snakes, bears, wolves, spiders, bats, birds, cats, ants, and beetles (see Supplementary material).

### Instrument 2: biophilia

To measure attraction to biodiversity, we created a non-verbal, image-based paired comparison task with three subdomains: Backyard Biophilia, Wildlife Tolerance, and Biophilic Travel. Following Felsten (2009), children selected between images representing biodiverse vs. non-biodiverse environments. The instrument was designed to minimize linguistic, age related and cultural bias, using printed photographs and pilot-tested imagery.

Backyard Biophilia trials contrasted pollinator gardens or woodlands with lawns and ornamental landscapes. Wildlife Tolerance trials featured wildlife-rich versus safe, familiar species near homes or in their neighborhood. Biophilic Travel trials compared biodiverse destinations (e.g., coral reefs, rainforests) with artificial or built environments (e.g., cities, pools). Selections of biodiverse options were coded as 1; scores ranged from 0 to 1 for each domain.

#### Pilot testing

The image-based task was pilot tested with 33 children aged 3–11 years at the North Carolina Zoo; 28 completed the task, and a random subsample of eight children also completed semi-structured interviews. Pilot testing identified image pairs in which children responded to unintended features of the images rather than biodiversity, as well as pairs that produced little variation in responses (>75% selection of one image). Eight image pairs were replaced across two days of testing, and five additional pairs were added to improve representation of the three biophilia domains. Pilot data were exploratory and were not included in the final analyses. Full details of the pilot study, image sets, selection criteria, and ecological diversity ratings are detailed in Supplementary material.

### Statistical analysis

All analyses were performed in SPSS v29. Shapiro–Wilk tests, skewness, kurtosis, and histogram inspections assessed normality of biophilia and biophobia scores (Prediction 1a), and we evaluated range dispersion to test for ceiling/floor effects (Prediction 1b). Shapiro–Wilk tests indicated departures from strict normality, as expected for bounded, discrete scores. However, skewness and kurtosis were modest and histogram inspection did not indicate severe departures from symmetry. Given the large sample sizes, parametric tests were retained for subsequent analyses, including independent-samples t-tests, Pearson correlations, and linear regression.

Cross-cultural differences between U. S. and Congolese children were tested using independent samples *t*-tests and multiple linear regression models, which included country, age, gender, and age × country interaction terms as predictors (Predictions 3a and 3b). To validate the structure of the biophilia instrument, we conducted an exploratory factor analysis using principal axis factoring with Promax rotation, based on *a priori* theoretical grouping into three domains: Backyard Biophilia, Wildlife Tolerance, and Biophilic Travel. Pearson’s correlation coefficients were used to examine the strength and direction of associations among the three biophilia domains (Prediction 4), both in the pooled sample and within each country.

Biophobia-biophilia associations (Prediction 5) were tested using Pearson’s correlations between fear scores and preference scores across domains. We also examined item-level correlations to assess specific animal fears versus biodiversity preferences.

To assess instrument sensitivity, we compared effect sizes for cultural differences across both instruments. A *post hoc* sensitivity analysis (Faul et al., 2009) indicated that a sample of 226 participants (113 per group) would provide 80% power to detect an effect of *d* = 0.375 at α = 0.05; our final sample exceeded this threshold. Supplementary material includes full demographic tables, image validation procedures, and detailed regression outputs.

## Results

### Prediction 1: individual variability in biophilia and biophobia

#### 1a. Distribution patterns

Biophilia and biophobia scores showed substantial individual variability in both samples. Shapiro-Wilk tests indicated departures from strict normality, as expected for bounded, discrete scores, although skewness and kurtosis were modest and histogram inspection did not indicate severe departures from symmetry.

#### 1b. Ceiling/floor effects

No substantial ceiling clustering was observed. Some lower-end clustering was present, particularly for Wildlife Tolerance and Biophilic Travel in the Congolese sample, although responses spanned the scale range in both populations. Mean biophobia scores were moderate (U. S. M = 0.64, Congo M = 0.69), and biophilia domain scores ranged from 0.3–0.5 across groups, indicating a range of responses without extreme clustering (Table 3).

**Table 3:** Means and normality indicators for biophobia and biophilic factors in U. S. and Congolese children.

Variable	Country	*N*	Mean	SD	95% CI [LL, UL]	Skewness (SE)	Kurtosis (SE)
Biophobia	US	124	0.64	0.43	[0.56, 0.71]	0.41 (0.22)	−0.13 (0.43)
	Congo	171	0.69	0.42	[0.63, 0.76]	0.41 (0.19)	0.07 (0.37)
Biophilia							
Backyard Biophilia	US	124	0.4	0.3	[0.37, 0.47]	0.43 (0.22)	−0.69 (0.43)
	Congo	171	0.4	0.3	[0.35, 0.43]	0.37 (0.19)	−0.54 (0.37)
Wildlife Tolerance	US	124	0.5	0.3	[0.46, 0.57]	−0.00 (0.22)	−0.88 (0.43)
	Congo	171	0.4	0.3	[0.36, 0.45]	0.09 (0.19)	−1.12 (0.37)
Biophilic Travel	US	124	0.5	0.3	[0.40, 0.52]	0.23 (0.22)	−0.94 (0.43)
	Congo	171	0.3	0.3	[0.24, 0.31]	0.76 (0.19)	−0.09 (0.37)

*N*, sample size; SD, standard deviation; CI, confidence interval; LL, lower limit; UL, upper limit. Skewness and kurtosis values include standard errors in parentheses.

### Prediction 2: early emergence

In the U.S. reference group, age positively predicted Wildlife Tolerance (*β* = 0.18, p = 0.046) and Biophilic Travel (*β* = 0.25, *p* = 0.005), but was not a significant predictor of Backyard Biophilia or biophobia. The full models explained a modest proportion of the variance, with *R^2^* = 0.05 for Wildlife Tolerance and *R^2^* = 0.13 for Biophilic Travel. These results suggest that some biophilic preferences may increase with age.

### Prediction 3: cross-cultural differences

#### 3a. Biophobia

Regression analyses indicated that country was a significant predictor of biophobia, with Congolese children reporting higher levels of fear toward animals than U. S. children (B = 0.16, SE = 0.07, *β* = 0.19, *p* = 0.03). However, the overall model explained only a small portion of the variance (R^2^ = 0.02, *F*(4, 290) = 1.44, *p* = 0.22), and age, gender, and the age × country interaction were not significant predictors (see Table 4).

**Table 4:** Multiple linear regressions predicting biophobia and biophilic outcomes from country, gender, age, and the country × age interaction.

Outcome (Model statistics)	Predictor	B	SE B	*β*	*t*	*p*
Biophobia (R^2^ = 0.020, *F*(4, 290) = 1.44, *p* = 0.220)	Country	0.16	0.07	0.19	2.26	.03
	Gender	−0.01	0.05	−0.01	−0.16	0.87
	Age	−0.01	0.01	−0.09	−0.91	0.36
	Country × Age	−0.03	0.03	−0.10	−1.17	0.24
Biophilia factors						
Wildlife Tolerance (R^2^ = 0.048, *F*(4, 290) = 3.64, *p* = 0.007)	Country	−0.17	0.05	−0.28	−3.48	<0.001
	Gender	−0.00	0.04	−0.00	−0.02	0.988
	Age	0.02	0.01	0.18	2.00	0.046
	Country × Age	−0.01	0.02	−0.04	−0.51	0.612
Backyard Biophilia (R^2^ = 0.038, *F*(4, 290) = 2.83, *p* = 0.025)	Country	−0.01	0.05	−0.01	−0.15	0.884
	Gender	0.09	0.03	0.16	2.78	0.006
	Age	0.01	0.01	0.05	0.49	0.624
	Country × Age	−0.02	0.02	−0.11	−1.32	0.189
Biophilic Travel (R^2^ = 0.128, *F*(4, 290) = 10.60, *p* < 0.001)	Country	−0.28	0.05	−0.46	−5.90	<0.001
	Gender	−0.04	0.03	−0.07	−1.27	0.204
	Age	0.03	0.01	0.25	2.84	0.005
	Country × Age	−0.01	0.02	−0.03	−0.31	0.756

Multiple linear regressions were conducted to predict biophobia and biophilic preferences from country (U. S. = 0, Congo = 1), gender (Male = 0, Female = 1), age (mean-centered), and the interaction between country and age. Significant predictors (*p* < 0.05) are highlighted in bold within the main text. Standardized coefficients (*β*) reflect the relative strength of each predictor.

However, item-level analyses revealed opposing fear patterns across the two groups. U. S. children were significantly more fearful of small arthropods and insects (e.g., spiders, ants, beetles), while Congolese children expressed more fear of larger predators and reptiles (e.g., bears, wolves, snakes, lizards). These divergent item-level patterns likely offset one another in the composite biophobia score, contributing to the null result in overall group comparison (see Figure 1).

**Figure 1:**
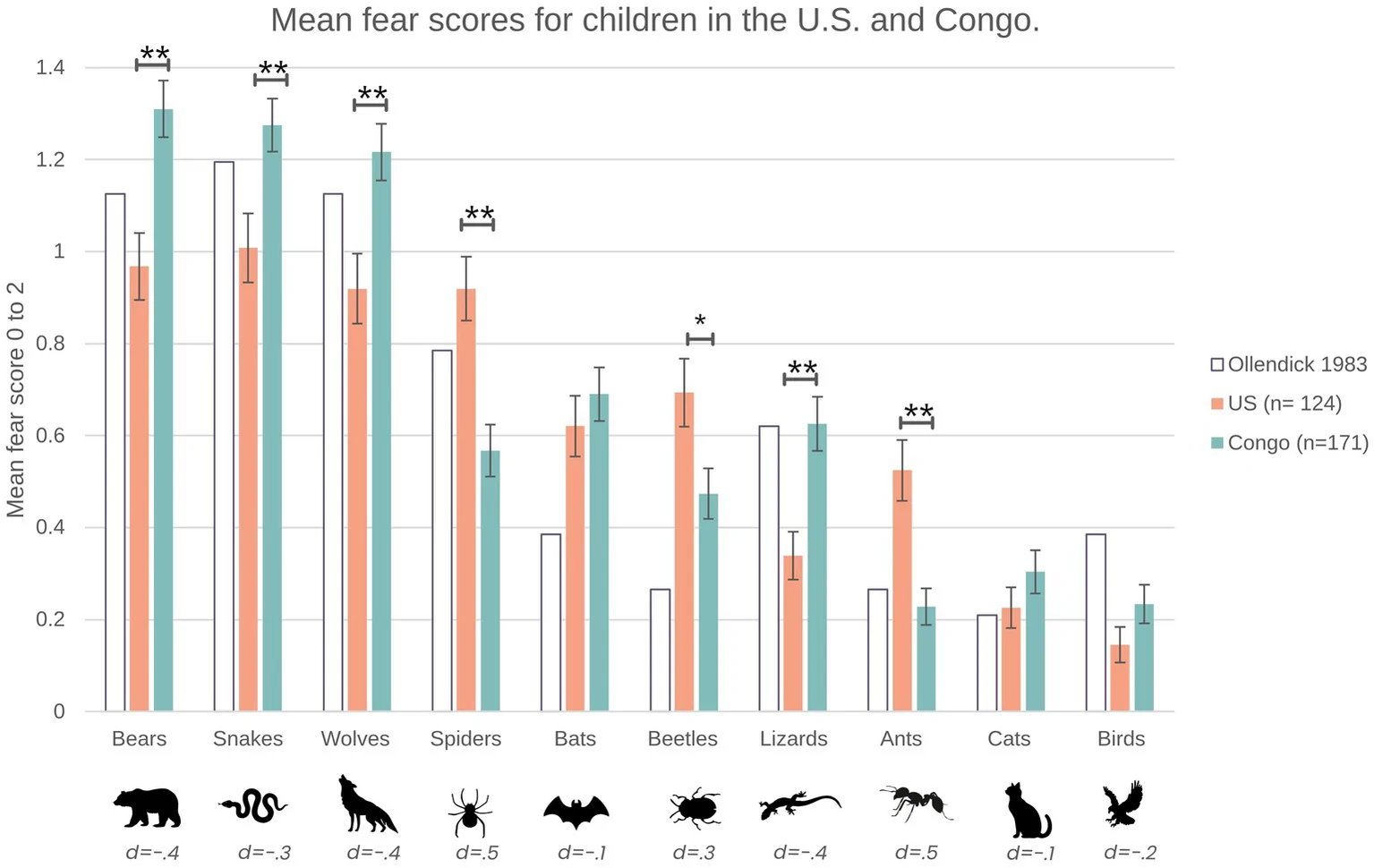
Mean fear scores for children in the U.S. and Congo. Mean fear scores for children in the United States (*n* = 124) and the Democratic Republic of Congo (*n* = 171) across 10 animal categories. Scores range from 0 (not afraid) to 2 (very afraid). Error bars represent standard errors. Asterisks indicate differences between the U.S. and Congolese samples from two-sided independent-samples t tests: one asterisk indicates *p* < 0.05; two asterisks indicate *p* < 0.01. Cohen’s d values appear beneath each animal category; negative values indicate higher fear in the Congolese sample, and positive values indicate higher fear in the U.S. sample. Historical comparison values from Ollendick (1983) were calculated by averaging the reported boys’ and girls’ means and subtracting 1 to express them on the 0-2 scale. The original combined items “bears or wolves,” “bats or birds,” and “ants or beetles” therefore share historical comparison values within each pair. See Supplementary Table A4 for full statistical results.

#### Biophilia

Country predicted Wildlife Tolerance (*β* = −0.28, *p* < 0.001) and Biophilic Travel (*β* = −0.46, *p* < 0.001), but not Backyard Biophilia. U. S. children had higher Wildlife Tolerance (M = 0.52 vs. 0.41) and Biophilic Travel (M = 0.46 vs. 0.28) scores than Congolese peers (*p* < 0.01). Backyard Biophilia showed no difference (*p* = 0.38).

Notably, mean scores across all biophilia domains were close to or below the scale midpoint (0.5), indicating that, on average, children in both countries expressed relatively modest biophilic preferences. This was especially striking in the U. S. sample, where children were visiting the zoo with their families—an activity typically associated with higher interest in nature. These mean scores suggest that even among a relatively nature-exposed group, strong biophilic preferences were not the norm.

#### 3b. Instrument sensitivity

To evaluate whether the preference-based biophilia instrument was more sensitive to cultural variation than the fear-based biophobia measure, we compared the predictive strength of country across models. In the biophobia model, country was a statistically significant predictor (B = 0.16, *β* = 0.19, *p* = 0.03), but the overall model explained minimal variance (R^2^ = 0.02). In contrast, country was a much stronger predictor of biophilia in two of the three domains: Wildlife Tolerance (WT) (B = −0.17, *β* = −0.28, *p* < 0.001, R^2^ = 0.05) and Biophilic Travel (B = −0.28, *β* = −0.46, *p* < 0.001, R^2^ = 0.13). No significant country effect was observed for Backyard Biophilia (B = −0.01, *p* = 0.88, R^2^ = 0.04).

### Prediction 4: internal consistency among domains

Exploratory factor analysis supported a three-factor structure consistent with theoretical domains (Table 5). Internal consistency for Wildlife Tolerance (α = 0.47) was modest. Correlations among biophilia domains were significant in the U. S. sample (*r* = 0.18–0.38), but weaker in the Congo sample. Cultural context influenced responses, particularly for Backyard Biophilia, where Congolese children sometimes preferred lawns for recreational or aspirational reasons.

**Table 5:** Exploratory factor analysis of two-choice paradigms.

Item	Factor loading		
	1	2	3
Factor 1: Biophilic Travel			
Q10. BT: Rainforest vs. Venice	**0.74**		
Q1. BT: Rainforest vs. Paris	**0.66**		
Q7. BT: Rainforest vs. Barcelona	**0.64**		
Q13. BT: Rainforest vs. indoor swimming pool	**0.49**	0.43	
Q4. BT: Coral reef vs. indoor swimming pool	**0.47**		
Factor 2: Wildlife Tolerance			
Q3. WT: Beetles vs. Butterflies		**0.71**	
Q6. WT: Bear, bats, wolf vs. deer		**0.69**	
Q15. WT: Reptiles incl. Snakes vs. lizards		**0.62**	
Q9. WT: Elephants, gorillas, leopards, vs. okapis	**0.36**	0.31	
Factor 3: Backyard Biophilia			
Q14. BB: Pollinator garden vs. lawn			**0.74**
Q11. BB: Pollinator garden vs. lawn			**0.69**
Q5. BB: Pollinator garden vs. formal garden	0.30		**0.41**
Q8 BB. Wildflowers vs. roses			**0.36**

*N* = 295. The extraction method was principal axis factoring with an oblique (Promax with Kaiser Normalization) rotation. Factor loadings above 0.30 are in bold, and if co-loaded, strongest value is in bold.

To further assess structural coherence, we examined correlations among the biophilia domains. Across the full sample, Wildlife Tolerance and Biophilic Travel were positively correlated (*r* = 0.30, *p* < 0.001), as were Backyard Biophilia and Biophilic Travel (*r* = 0.17, *p* = 0.003). Backyard Biophilia and Wildlife Tolerance were not significantly correlated (*r* = 0.06, *p* = 0.282). When split by country, all three domains were significantly correlated in the U. S. sample, whereas in the Congolese sample, only Wildlife Tolerance and Biophilic Travel were significantly associated (see Table 6). These findings support the domain-general nature of biophilic preference, though cultural context appeared to influence the strength and pattern of these relationships.

**Table 6:** Correlations among Wildlife Tolerance, Backyard Biophilia, and Biophilic Travel in U. S. and Congolese children.

Variables	1. BB	2. WT	3. BT
U. S. sample (*N* = 124)			
1. Backyard Biophilia (BB)	—	0.18*	0.21*
2. Wildlife Tolerance (WT)		—	0.38**
3. Biophilic Travel (BT)			—
Congolese sample (*N* = 171)			
1. Backyard Biophilia (BB)	—	−0.05	0.12
2. Wildlife Tolerance (WT)		—	0.16*
3. Biophilic Travel (BT)			—

Pearson correlation coefficients are reported. BB, Backyard Biophilia; WT, Wildlife Tolerance; BT, Biophilic Travel. *p < 0.05 (2-tailed). **p < 0.01 (2-tailed).

In particular, responses to Backyard Biophilia in the Congolese sample often reflected values tied more to play than to biodiversity. One 13-year-old boy noted, “I can play, ride my bicycle. The other place does not have enough space, there are too many flowers,” while an 11-year-old girl remarked, “I love the lawn, I would play with my dolls.”

### Prediction 5: relationship between biophilia and biophobia

Although the Biophilia Reactivity Hypothesis proposed that biophilia and biophobia would lie at opposite ends of a single continuum, our findings did not support this assumption. Biophobia was not significantly correlated with any of the three biophilia domains in either sample, indicating that low fear does not necessarily predict high attraction to biodiversity.

In the U. S. sample, biophobia showed no significant associations with Backyard Biophilia (*r* = 0.13, *p* = 0.15), Wildlife Tolerance (*r* = 0.00, *p* = 0.999), or Biophilic Travel (*r* = −0.07, *p* = 0.45). Similarly, among Congolese children, correlations between biophobia and Backyard Biophilia (*r* = 0.09, *p* = 0.25), Wildlife Tolerance (*r* = 0.01, *p* = 0.89), and Biophilic Travel (*r* = 0.03, *p* = 0.66) were negligible and nonsignificant.

To explore this dissociation further, we conducted item-level analyses linking fear of individual animals (e.g., snakes, bats, wolves) to choices in the Wildlife Tolerance task, where children selected between less diverse, non-threatening animal groups and more biodiverse scenes that included feared species. However, fear ratings did not predict biodiversity preferences. For example, children who reported fear of beetles were no less likely to choose the image containing butterflies and beetles (*r* = 0.063, *p* = 0.284, n = 295; see Figure 2).

**Figure 2:**
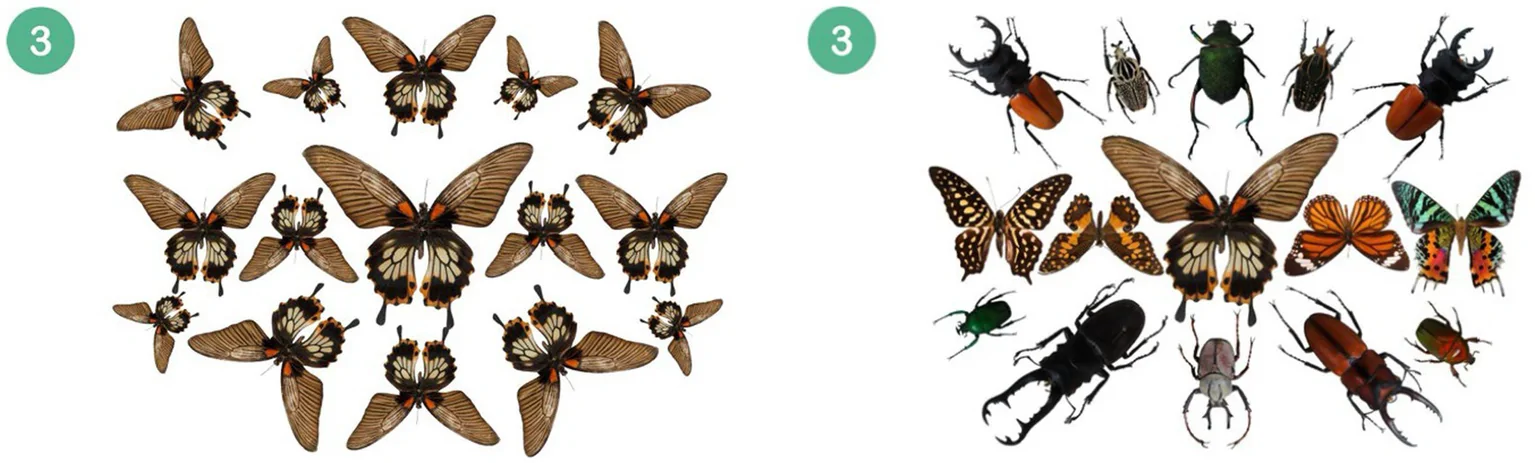
Example of paired sample of wildlife tolerance. In this Wildlife Tolerance trial, children were asked, “Who would you invite into your backyard?”. Children chose between a less diverse insect group and a more biodiverse image including beetles and butterflies. Fear of beetles did not predict choice: children who reported beetle fear were equally likely to select the biodiverse option. Adapted from iStock.com/katylaveckphotography, image 668850302, used under license. This third-party material is excluded from the article’s Creative Commons license.

Across both samples, fear of individual animals did not predict avoidance of biodiversity-rich scenes containing those animals (|*r*| < 0.12, ps > 0.14; see Supplementary material).

This pattern was reflected qualitatively. One Congolese girl who reported fear of 7 out of 10 animals on the biophobia scale nonetheless said: “I like to visit the forest. There are trees to protect me.” Another child who was afraid of four animals stated, “I find it beautiful… the trees… there are animals here and I love animals.” These examples reinforce the view that fear and attraction can coexist in the same individual.

Taken together, these findings suggest that biophilia and biophobia operate as distinct motivational systems: biophobia reflects aversive responses to specific animal threats, while biophilia captures a broader orientation toward biodiversity and natural environments (see Supplementary material for full statistical details, regression outputs, EFA matrices, and illustrative quotes).

## Discussion

Our findings provide empirical support for several predictions of the Biophilia Reactivity Hypothesis. Children showed substantial individual variability and early-emerging differences in biophilic preferences, cross-cultural differences were evident, and biophobia emerged as a distinct, non-inverse construct.

Across both samples, biophilia and biophobia scores showed substantial individual variability. Although the bounded, discrete measures departed from strict normality and some lower-end clustering was present, scores varied broadly across children. This provides evidence for the first criterion of a temperament trait, individual variability. The second criterion of a temperament trait is early emergence. Even at a young age, children varied in their preferences for biodiversity in the home garden, the level of biodiversity they would welcome in and around their neighborhoods, and the biophilic appeal of travel destinations. For example, a 13-year-old Congolese boy described the forest as “beautiful,” especially in contrast to urban settings: “The other place has too many people, too many cars.” In contrast, an 8-year-old American girl said: “I like the city because that’s somewhere I can go to the bathroom, and I would not get lost.”

Most of the younger participants were from the U. S., offering insight into early developmental variability. One 5-year-old boy expressed strong natural affinity, saying, “You know why I keep [choosing] forest, forest, forest? I love a forest!” In contrast, a 4-year-old preferred city environments because “Mom can drive me around.” When asked to choose between biodiverse pollinator gardens and non-biodiverse lawns, one child preferred the garden because “It’s pretty with lots of flowers,” while another chose lawns, explaining, “I could let my dogs play here.”

Across both groups, mean scores across the biophilia domains were close to or below the midpoint of the scale, suggesting that strong biophilic preferences are not the default in childhood. This was surprising given that both groups were visiting a zoo or a sanctuary—settings typically expected to prime interest in biodiversity. This was especially unexpected in the U. S. sample, where children visited the zoo with their families and might have had an intrinsic desire to be there (although parents or siblings may have influenced the visit). Because these contexts are likely to elevate biophilic responding, if anything, the scores observed here may be artificially high. Future research should examine biophilic responses in more neutral environments to better assess baseline orientations toward biodiversity.

Even so, cross-cultural analyses revealed robust differences in biophilic preferences, with Congolese children scoring lower in their tolerance for wildlife in their neighborhoods, and desire to travel to biodiverse locations. These children, despite living in a highly biodiverse country, often remarked on the safety, cleanliness, and esthetic attraction of cities. For example, an 11 year old girl said “the city is very pretty… the other place might have a crocodile.” These differences were statistically significant. These differences emphasize the importance of not only including data from non-Western countries, but examining the ecological context within countries. Notably, all Congolese children in this study lived in Kinshasa, a rapidly growing megacity with substantial biodiversity loss and limited daily exposure to wildlife.

This pattern underscores the importance of how biodiversity preferences are measured. While the biophobia scale’s composite score masked meaningful group-level variation, the biophilia instrument—particularly the Biophilic Travel and Wildlife Tolerance subscales—was more sensitive to cross-cultural differences. These scales not only produced stronger effect sizes but also explained more variance, suggesting that preference-based assessments may better detect cultural patterns in children’s orientation toward biodiversity.

Factor analysis further supported the structure of the biophilia instrument, revealing three coherent domains: Backyard Biophilia, Wildlife Tolerance, and Biophilic Travel. Wildlife Tolerance and Biophilic Travel were moderately correlated across the full sample, suggesting a shared underlying attraction to biodiversity—even when that biodiversity may include potentially fear-inducing species. Backyard Biophilia was less strongly related, particularly in the Congolese sample, where it was not significantly associated with the other two domains. This may reflect the role of socioeconomic context: in Kinshasa, lawns and managed gardens often require domestic help, water access, and land ownership, privileges typically limited to wealthier households. Preferences for backyard settings may therefore reflect social aspiration as much as environmental affinity.

Crucially, we found no evidence that fear predicts lack of attraction to biodiversity. In contrast with previous work with adults (Woods et al., 2026), biophobia was not significantly correlated with any biophilia domain in either sample, indicating that fear and attraction may operate independently.

Item-level analyses further confirmed this dissociation. In the Wildlife Tolerance preference tests, children were presented with the choice of inviting a group of less diverse, non-threatening species into the area where they lived and more biodiverse scenes that included animals from the biophobia scale. Fear ratings did not predict preference patterns. These examples show that children can simultaneously experience fear and attraction, and that biophilia cannot be inferred from the absence of biophobia.

In sum, this study supports the Biophilia Reactivity Hypothesis. Biophilia appears to be a temperament-like trait marked by early-emerging, individually variable attraction toward biodiversity. Biophobia and biophilia function as distinct systems: one is the fear of individual animals; the other, attraction to biodiversity.

This reframing has important theoretical and practical implications. First, it offers a plausible explanation for the long-observed attitude-behavior gap in conservation psychology: if only some individuals are attracted to biodiversity, then universal appeals may fail to resonate broadly. Second, it provides a more nuanced model for understanding how biophilic tendencies develop, persist, and translate into conservation-relevant behaviors. In addition, the measurement tools developed here may offer a valuable framework for evaluating biodiversity education and nature-based interventions. By capturing individual variation in biophilic preferences, they allow for indirect tests of whether such programs shift children’s orientation toward biodiversity—particularly in settings where long-term behavioral change is difficult to assess.

Future research should explore the remaining two criteria of a temperament trait; consistency over time and physiological markers. Also, while this study sampled Congolese children living in Kinshasa, an urban setting characterized by rapid deforestation and reduced daily exposure to wildlife, future work should aim to include children who live in closer proximity to the biodiversity at the heart of the biophilia hypothesis—such as those residing within or adjacent to the Congo Basin. These populations may exhibit meaningful differences to urban populations.

Rather than being universal or innate, biophilia varies in strength and form—calling for more nuanced, domain-specific, and culturally informed approaches to measuring human orientation toward the natural world.

## Conclusion

The central question of this study was whether children’s attraction to biodiversity is better characterized as a human universal or a temperament trait. The findings support the Biophilia Reactivity Hypothesis on the two temperament criteria tested here: individual variability and early emergence. Biophilic preferences varied substantially among children in both countries and were evident from early childhood. At the same time, biophobia was not the inverse of biophilia, suggesting that attraction to biodiversity and fear of animals are distinct. The remaining two criteria of a temperament trait, stability over time and physiological signature, should be tested in future research.

Future studies should incorporate observational, behavioral, and physiological measures, alongside preference tasks, to further assess trait-level biophilia. Integrating data across diverse cultural settings, developmental stages, and biodiversity contexts will be critical to validate biophilia as a temperament trait and to inform interventions tailored to individual dispositions and ecological conditions. By moving toward a developmentally sensitive, culturally grounded, and ecologically valid model, we can improve the accuracy of biophilia assessments and the effectiveness of conservation engagement strategies for the next generation.

In conclusion, this study provides empirical support for the Biophilia Reactivity Hypothesis and lays the groundwork for testing biophilia as a measurable, multidimensional, and culturally modulated temperament trait. By moving beyond universals to embrace individual variability, we may be better equipped to promote effective biodiversity engagement and conservation behavior in the next generation.

## Data Availability

The datasets generated and analyzed for this study are not publicly available due to concerns regarding participant anonymity because the study involved children. De-identified data supporting the conclusions of this article are available from the corresponding author upon reasonable request, subject to applicable ethical and privacy requirements. Study materials, including the survey instruments and image-based tasks, are also available from the corresponding author upon reasonable request.
