# Supplementary material for: Biophilia as a temperament trait, not a universal: children’s attraction and aversion to biodiversity in the United States and Democratic Republic of Congo
Source: Front Psychol. 2026 Oct 30;17:1904601. doi: 10.3389/fpsyg.2026.1904601 (PMC13759342; doi:10.3389/fpsyg.2026.1904601)
Supplement: Supplementary file 1 [file Supplementary_file_1.docx]

## Supplemental A

### **Supplemental Methods**

### **Pilot Study**

#### **Participants**

The pilot study involved 33 children aged 3 to 11 years, recruited at the North Carolina Zoo between May 8 and May 10, 2024. Five children did not complete the testing session, resulting in a final sample of 28 participants (see Table A1 for age distribution). Semi-structured interviews were conducted with a random sample of 8 children.

#### **Image Changes and Replacements**

During pilot testing, responses were reviewed for consistency and accuracy. Four pairs were replaced on the first day due to unintended elements (e.g., “a basketball would hit you”) or a lack of variation in responses (>75% selection). On the second day, another four pairs were replaced for similar reasons. Five additional pairs were introduced to increase category representation, including comparisons of lawns versus pollinator gardens and rainforest versus urban scenes. The pilot data were exploratory and were not included in the final analyses.

##### Table A1. Demographic Characteristics of Pilot Study Participants

| **Characteristic** | **n** | **%** |
| --- | --- | --- |
| **Gender** |  |  |
| F | 22 | 66.7 |
| M | 11 | 33.3 |
| **Age** |  |  |
| 3 | 1 | 3 |
| 4 | 4 | 12.1 |
| 5 | 3 | 9.1 |
| 6 | 3 | 9.1 |
| 7 | 4 | 12.1 |
| 8 | 8 | 24.2 |
| 9 | 3 | 9.1 |
| 10 | 2 | 6.1 |
| 11 | 5 | 15.2 |
| Included in analysis | 28 | 84.8 |
| Excluded (any ND) | 5 | 15.2 |
| Total | 33 | 100 |

*Note.* The table summarizes gender and age distributions for the full pilot sample (N = 33) and notes the final analytic sample (n = 28) after excluding five participants who did not complete testing (i.e., provided no data on more than 1 item).

#### **Image Selection Procedures**

A total of 60 images were curated to represent three factors: Wildlife Tolerance, Backyard Biophilia, and Biophilic Travel. Each factor included 20 images, organized into 10 pairs evenly divided between biodiverse and non-biodiverse settings. Images were required to exceed 1 MB in size and have a resolution of at least 72 dpi, and were captured at eye level under favorable weather conditions, adhering to criteria from Han (2007).

**Backyard Biophilia:** Images contrasted biodiverse yards and gardens (e.g., pollinator gardens with >80% woodland or native plant coverage) with non-biodiverse settings (e.g., traditional lawns and non-native shrub landscaping). Some images included age-appropriate activities, such as soccer or playgrounds.

**Wildlife Tolerance:** Twenty-nine images were initially evaluated by a panel of four experts and 13 undergraduates using the Delphi method*^44^*. After revisions based on panel recommendations, 12 images were pilot tested, leading to the removal of four items. The final set included eight images (four pairs), depicting choices between biodiverse wildlife groups (including species from the FSSC-R list, e.g., bears, wolves, beetles) and less biodiverse, safer alternatives. One paired set featured U.S. wildlife; another featured Congolese wildlife.

**Biophilic Travel:** Images depicted biodiverse tropical rainforests and coral reefs versus non-biodiverse urban environments or chlorinated swimming pools. Species composition and relative abundance were estimated via digital photograph analysis and supported by a large language model (ChatGPT, OpenAI) to calculate diversity indices and generate visual field estimates. Green space estimates and reef biodiversity cover were derived using pixel-based image analysis calculations conducted via OpenAI's GPT-4, which includes multimodal image interpretation capabilities. All estimates were confirmed by two authors.

### **Table A2. Characteristics of visual stimuli used in the biophilia task**

**Note.** Original stimulus photographs are not reproduced because they are third-party images. Text descriptions refer to the images presented to participants during testing; no substitute images are shown. Images can be supplied upon request to the author.

#### Backyard Biophilia

| Stimulus description | Species number | % vegetation | Shannon–Wiener Index (H′) |  |
| --- | --- | --- | --- | --- |
| Woodland playground beneath mature trees | 6 | 60 | 1.39 | American Beech (*Fagus grandifolia*) – 6 individuals – 40%  Oak (*Quercus* sp.) – 1 individual – 10%  Poison Ivy (*Toxicodendron radicans*) – 30%  Understory Shrubs (unidentified) – 15%  Vines/Saplings (unidentified) – 5% |
| Playground on mown lawn with sparse background trees/shrubs | 4 | 60 | 1.39 | **Mown Lawn Grass Estimated % coverage:** ~85% **Species count:** 1  **Distant Trees / Shrubs** *(blurred background),* **Estimated % coverage:** ~10% **Species count:** ~3 (broad estimate due to image blur) |
| Formal geometric garden with clipped hedges and lawn pathways | 4 | 95% | 1.19 | Light green hedges – 1 species – 45% cover  Gray-green hedges (center of shapes) – 1 species – 30% cover  Background hedge (tall, dense, dark green wall) – 1 species – 20% cover Grass lawn (pathways) – 1 species – 5% cover |
| Informal flower-rich garden with bench and mixed vegetation | 8-12 | 90% | 1.83 | Nettle (yellow flowers) – 1 species – 20% cover  Dianthus (pink flowers) – 1 species – 10% cover  Other pink flowers – 1 species – 10% cover Hemlock (conifer) – 1 species – 10% cover  Purple flowers – 1 species – 10% cover  Ground cover (mixed herbs) – 2–3 species – 30% cover  Vine on bench – 1 species – 10% cover |
| Diverse mixed wildflower planting | 9 | 100 | 2.11 | **California poppies (orange/yellow)** – 25%  **Calendula (orange)** – 10% **Red poppies** – 10%  **Cosmos (red/pink)** – 10%  **Light pink flowers** – 10%  **Cornflowers (blue)** – 10%  **Pink flowers (species 1)** – 10%  **Pink flowers ( species 2)** – 10%  **Purple flowers** – 5% |
| Rose-dominated flower garden | 1 | 100 | 0 | Rose sp |
| Mown lawn bordered by ornamental shrubs and flowers | 7 | 75 | 1.13 | **Lawn Grass** – 1 species – 70% cover  **Boxwood or Low Shrubs** – 1 species – 8% cover  **Peony (pink flowering shrub)** – 1 species – 4% cover  **Daylilies (sprouting foliage near mulch)** – 1 species – 4% cover  **Conifers (small evergreen shrubs/trees)** – 1 species – 5% cover  **Azaleas (flowering shrubs along back fence)** – 1 species – 6% cover  **Weeping Cherry Tree** – 1 species – 3% cover |
| Mixed flowering garden with high plant diversity | 16 | 90 | 2.63 | **Salvia (purple flower spikes)** – 1 species – 15% cover  **Echinacea (pink coneflowers)** – 1 species – 10% cover  **Phlox (tall pink flowers)** – 1 species – 10% cover  **Fleabane (white daisy-like flowers)** – 1 species – 5% cover  **Joe Pye Weed (tall pink clusters)** – 1 species – 5% cover  **Persicaria (red flower spikes)** – 1 species – 5% cover  **Red flower species (additional)** – 2 species – 10% cover total **Common Milkweed (Asclepias syriaca)** – 1 species – 5% cover  **Mountain Mint (Pycnanthemum spp.)** – 1 species – 10% cover  **Grasses (various)** – 1 species – 10% cover  **Broad-leaved foliage plants (non-flowering)** – 1 species – 5% cover  **Other herbaceous plants (background/edge)** – 1 species – 5% cover **Mature Trees (various species)** – 4 species – <1% cover each |
| Dense pollinator-style mixed flower garden | 14 | 100 | 2.64 | **California poppy** – 5%  **Orange calendula** – 5%  **Red poppy** – 7% **Red/pink cosmos** – 6% **Blue cornflower** – 8% **Purple salvia** – 7% **Phlox** – 7%  **Tall red snapdragon** – 10%  **Foxglove** – 4%  **Fleabane** – 5%  **Pink geranium** – 6%  **Lupine** – 8%  **Japanese maple** – 12%  **Unknown pink flower #1** – 5%  **Unknown pink/purple flower #2** – 5% |
| Manicured lawn bordered by shrubs and groundcover | 5 | 95 | 0.47 | Turfgrass (lawn) – 1 species – 85% cover  Shrubs (mid-ground, 2 types) – 2 species – 5% cover  Tall hedge (background) – 1 species – 3% cover  Groundcover (black-leaved plant) – 1 species – 2% cover |

#### Wildlife Tolerance

| Stimulus description | Species number | Total number | Shannon–Wiener Index (H') | Pielou’s Evenness (J') |
| --- | --- | --- | --- | --- |
| Deer-dominated assemblage with familiar small mammals | 11 | 20 | 1.94 | 0.81 |
| More diverse mammal assemblage including deer, large mammals, and small mammals | 19 | 24 | 2.87 | 0.97 |
| Mixed insect assemblage including beetles and butterflies/moths | 13 | 14 | 2.54 | 0.99 |
| Butterfly/moth assemblage | 1 | 16 | 0 | 0 |
| Diverse African wildlife assemblage including large mammals | 22 | 22 | 3.09 | 1.0 |
| Less-diverse, okapi-dominated wildlife assemblage | 13 | 23 | 1.99 | 0.74 |
| Diverse reptile/amphibian assemblage | 15 | 21 | 2.62 | 0.97 |
| Single-species lizard assemblage | 1 | 17 | 0 | 0 |

#### Biophilic Travel

|  | **% greenspace** | **Description** |
| --- | --- | --- |
| Tropical rainforest landscape with waterfall | 90–95% | **Very high green cover**, typical of tropical rainforest landscapes. |
| Urban boulevard/cityscape with trees | 15–20% | L**ow green cover**, though it reflects a relatively greener section of an urban setting thanks to planned tree-lined boulevards. |
| Dense tropical forest scene with wildlife | 85–90% | **Very high green cover**, consistent with dense tropical forest imagery. |
| Urban street with buildings and concentrated vegetation | 25–30% | **moderate green cover** in a built environment with concentrated but limited greenery. |
| Urban canal/cityscape with limited vegetation | 20–25% | L**ow-to-moderate green cover** for an urban landscape. |
| Mature tropical rainforest/forest river landscape | 85–90% | **Very high green cover**, typical of a mature, tropical rainforest scene |
| Indoor/resort-style swimming pool with sparse plants | 5–10% | Very low green cover; indoors with a few sparse palms and plants |
| Indoor lap swimming pool with no visible vegetation | 0% | No biodiversity |
| Coral reef scene with fish, coral, and reef structure | 40–50% | High coral reef presence including coral formations, anemones, and reef structures |

#### **Demographic Characteristics**

##### Table A3. Full characteristics of children who participated stratified by country, gender, and age, including missing data

|  |  | **TOTAL** | |  |  | **US** |  |  |  | **Congo** | |  |
| --- | --- | --- | --- | --- | --- | --- | --- | --- | --- | --- | --- | --- |
|  |  | (n=310) | |  |  | (n=133) | |  |  | (n=177) | |  |
|  |  |  |  |  |  |  |  |  |  |  |  |  |
| **Sample characteristic** | n | ND | % | ND | n | ND | % | ND | n | ND | % | ND |
| **Demographics** | 295 | 15 | 95% | 5% | 124 | 9 | 93% | 7% | 171 | 6 | 97% | 3% |
|  |  |  |  |  |  |  |  |  |  |  |  |  |
| **Gender** |  |  |  |  |  |  |  |  |  |  |  |  |
| Male | 139 | 6 | 45% | 2% | 57 | 6 | 43% | 5% | 82 | 0 | 46% | 0% |
| Female | 156 | 9 | 50% | 3% | 67 | 3 | 50% | 2% | 89 | 6 | 50% | 3% |
|  |  |  |  |  |  |  |  |  |  |  |  |  |
| **Age, years** |  |  |  |  |  |  |  |  |  |  |  |  |
| 3 | 12 | 3 | 4% | 1% | 12 | 3 | 9% | 2% | 0 | 0 | 0% | 0% |
| 4 | 11 | 1 | 4% | 0% | 11 | 1 | 8% | 1% | 0 | 0 | 0% | 0% |
| 5 | 16 | 3 | 5% | 1% | 15 | 3 | 11% | 2% | 1 | 0 | 1% | 0% |
| 6 | 20 | 3 | 6% | 1% | 20 | 2 | 15% | 2% | 0 | 1 | 0% | 1% |
| 7 | 11 | 0 | 4% | 0% | 9 | 0 | 7% | 0% | 2 | 0 | 1% | 0% |
| 8 | 21 | 1 | 7% | 0% | 16 | 0 | 12% | 0% | 5 | 1 | 3% | 1% |
| 9 | 25 | 0 | 8% | 0% | 14 | 0 | 11% | 0% | 11 | 0 | 6% | 0% |
| 10 | 61 | 0 | 20% | 0% | 14 | 0 | 11% | 0% | 47 | 0 | 27% | 0% |
| 11 | 51 | 0 | 16% | 0% | 3 | 0 | 2% | 0% | 48 | 0 | 27% | 0% |
| 12 | 51 | 0 | 16% | 0% | 7 | 0 | 5% | 0% | 44 | 0 | 25% | 0% |
| 13 | 16 | 0 | 5% | 0% | 3 | 0 | 2% | 0% | 13 | 0 | 7% | 0% |
| >14 | 0 | 4 | 0% | 1% | 0 | 0 | 0% | 0% | 0 | 4 | 0% | 2% |

Abbreviations: ND= missing or incomplete data

### **Supplemental Results**

#### **Biophobia Score Distribution**

Figure A1 illustrates the distribution of biophobia scores among children in the United States and the Democratic Republic of Congo. Biophobia scores reflect the average level of aversive response to animals, assessed across multiple trials, with possible values ranging from 0 (no fear) to 2 (high fear). The histogram shows the percentage of children in each country corresponding to each mean score, while the overlaid dashed lines represent normal curve approximations for each group. The distributions show substantial individual variability in both samples. Although the overlaid dashed lines provide normal-curve approximations, Shapiro-Wilk tests indicated departures from strict normality. This pattern supports the prediction of individual variability in biophobia and suggests that strong fear responses to animals are not universally elevated in either cultural context.

Figure A1. Histogram of biophobia scores in U.S. and Congolese children

*Note. Bars represent the percentage of children in each country with a given mean biophobia score (range: 0–2). Dashed lines indicate normal curve approximations for each group. Biophobia reflects aversive responses to animals across multiple trials.*

Figure A2 displays the distributions of biophilia scores across the three domains measured in the study. Each histogram represents children’s average scores within a domain, ranging from 0 to 1. Dashed lines indicate normal-curve approximations for each factor. Scores showed substantial individual variability across domains. Shapiro-Wilk tests indicated departures from strict normality, and some lower-end clustering was present, particularly for Wildlife Tolerance and Biophilic Travel in the Congolese sample.

Figure A2. Distribution of biophilia factors

#### Independent Samples t-Tests

Figure A3 presents the results of independent samples t-tests comparing biophilia scores between children in the United States and the Democratic Republic of Congo across three domains: Wildlife Tolerance, Backyard Biophilia, and Biophilic Travel. Mean scores for each group are displayed with 95% confidence intervals, and asterisks denote statistically significant differences (p < .01). Effect sizes (Cohen’s d) are reported beneath each domain, indicating the magnitude of group-level differences. U.S. children showed significantly higher biophilia scores in the Wildlife Tolerance and Biophilic Travel domains, while no significant difference was observed in Backyard Biophilia. These results highlight both cultural variability in biophilic preferences and the importance of instrument sensitivity in detecting such differences.

##### Figure A3. Independent samples t test of biophilic factors in children in the United States and Congo.

Note: Mean Wildlife Tolerance, Backyard Biophilia, and Biophilic Travel scores for children in the United States and Congo. Error bars represent 95% confidence intervals. Asterisks indicate statistically significant differences between countries (*p* < .01). Effect sizes (Cohen’s *d*) are reported below each factor.

#### Biophobia Results

Table A4 presents the results of independent samples t-tests comparing fear responses to individual animals between children in the United States and the Democratic Republic of Congo. For each animal, mean fear scores and standard deviations are reported by country, along with test statistics, p-values, confidence intervals for the mean difference, and Cohen’s d effect sizes. Negative t and Cohen’s d values indicate higher fear in the Congolese sample, while positive values reflect higher fear in the U.S. sample. Statistically significant differences (p < .05) are bolded. U.S. children reported significantly more fear of spiders, ants, and beetles, while Congolese children showed significantly higher fear of larger mammals and reptiles, such as bears, snakes, wolves, and lizards. These results reveal both cross-cultural differences in specific animal fears and broad variability in biophobic responses.

##### Table A4. Independent samples t-test results for fear of individual animals in U.S. and Congolese children

| **Animal** | **M (US)** | **SD (US)** | **M (Congo)** | **SD (Congo)** | **t(df)** | **p** | **95% CI (Mean Diff)** | **Cohen's d [95% CI]** |
| --- | --- | --- | --- | --- | --- | --- | --- | --- |
| Bears | 1.0 | 0.8 | 1.3 | 0.8 | -3.58 (293) | **< .001** | [-0.53, -0.15] | -0.42 [-0.66, -0.19] |
| Snakes | 1.0 | 0.8 | 1.3 | 0.8 | -2.86 (293) | **.005** | [-0.45, -0.08] | -0.34 [-0.57, -0.10] |
| Wolves | 0.9 | 0.9 | 1.2 | 0.8 | -3.05 (293) | **.003** | [-0.49, -0.11] | -0.36 [-0.59, -0.13] |
| Spiders | 0.9 | 0.8 | 0.6 | 0.7 | 3.95 (293) | **< .001** | [0.18, 0.53] | 0.47 [0.23, 0.70] |
| Bats | 0.6 | 0.7 | 0.7 | 0.8 | -0.78 (293) | .434 | [-0.24, 0.10] | -0.09 [-0.32, 0.14] |
| Beetles | 0.7 | 0.8 | 0.5 | 0.7 | 2.43 (293) | **.016** | [0.04, 0.40] | 0.29 [0.05, 0.52] |
| Ants | 0.5 | 0.7 | 0.2 | 0.5 | 4.04 (293) | **< .001** | [0.15, 0.44] | 0.48 [0.24, 0.71] |
| Lizards | 0.3 | 0.6 | 0.6 | 0.8 | -3.50 (293) | **< .001** | [-0.45, -0.13] | -0.41 [-0.65, -0.18] |
| Cats | 0.2 | 0.5 | 0.3 | 0.6 | -1.17 (293) | .242 | [-0.21, 0.05] | -0.14 [-0.37, 0.09] |
| Birds | 0.1 | 0.4 | 0.2 | 0.5 | -1.50 (293) | .135 | [-0.21, 0.03] | -0.18 [-0.41, 0.06] |

***Note.*** *Means (M) and standard deviations (SD) are reported separately for U.S. (n = 124) and Congolese (n = 171) participants. Tests are two-sided independent-samples t tests assuming equal variances, with 293 degrees of freedom. Mean differences are calculated as U.S. minus Congo. Negative t and Cohen’s d values indicate higher fear in the Congolese sample; positive values indicate higher fear in the U.S. sample. Statistically significant p values (p < .05) are bolded. CI = confidence interval.*

An independent samples t-test further revealed no significant difference in overall biophobia scores between U.S. (M = 0.64, SD = 0.43) and Congolese (M = 0.69, SD = 0.42) children, t(293) = –1.13, p = .26, with a small and nonsignificant effect size (Cohen’s d = –0.13).

**Unique Capture of Attraction**: To assess whether the biophilia instrument uniquely captured active attraction to biodiversity rather than simply reflecting low levels of fear, we examined the associations between biophobia and each biophilia domain. As reported in Prediction 5, none of the biophilia domains were significantly correlated with biophobia in either the U.S. or Congolese samples. Correlation coefficients were small and nonsignificant across all pairings (e.g., Wildlife Tolerance–Biophobia, U.S.: *r* = .000, *p* = .999; Congo: *r* = .011, *p* = .890), suggesting that fear-based responses and preference-based engagement operate as independent constructs.

Furthermore, country-level differences were robust in biophilia (Wildlife Tolerance and Biophilic Travel), but not in biophobia. Taken together, these findings support the conclusion that the preference-based biophilia instrument captures dimensions of attraction that cannot be inferred from the absence of fear alone, reinforcing its validity as a distinct and necessary tool for assessing children's orientation toward biodiversity.

### Correlational Analyses

*Correlations Between Biophobia and Biophilia Factors.*

Table A5 reports Pearson correlations between biophobia and three biophilic factors—Backyard Biophilia, Biophilic Travel, and Wildlife Tolerance—in children from the United States and the Democratic Republic of Congo. Correlation coefficients (r) and associated p-values are provided for each relationship. In both samples, biophobia was not significantly correlated with any of the biophilic domains, suggesting that fear of animals and attraction to biodiversity are largely independent constructs in early development. These findings support the hypothesis that biophobia and biophilia operate along distinct dimensions, rather than existing as opposite ends of a single continuum.

##### Table A5. Correlations Between Biophobia and Biophilia Factors

| Variable | Backyard Biophilia (r, p) | Biophilic Travel (r, p) | Wildlife Tolerance (r, p) | |
| --- | --- | --- | --- | --- |
| U.S. sample (N = 124) |  |  | |  |
| Biophobia | .131 (.146) | –.068 (.454) | | .000 (.999) |
| Congolese sample (N = 171) |  |  | |  |
| Biophobia | .089 (.247) | .034 (.661) | | .011 (.890) |

Note. Pearson correlations are reported. Values are presented as r (p-value). *p < .05. **p < .01.

**Exploratory item-level correlations**

Table A6 reports exploratory Pearson correlations between selected Wildlife Tolerance items and item-level animal fear scores, separately by country. These analyses were descriptive and were not used to define the biophilia domains. No significant associations emerged, and most correlations were near zero.

##### Table A6. Exploratory item-level correlations

| Variables | US r | US p | Congo r | Congo p |
| --- | --- | --- | --- | --- |
| WT- bug group x beetle fear | .071 | .432 | .02 | .793 |
| WT-deer group × Bats fear | .049 | .589 | –.023 | .769 |
| WT-okapi group × Snakes fear | –.038 | .673 | .031 | .690 |
| WT-deer group × Bears fear | –.024 | .793 | –.111 | .150 |
| WT-deer group × Wolves fear | –.067 | .463 | –.065 | .399 |
| WT Q15 × Snakes fear | –.069 | .448 | –.004 | .961 |

**Note.** Pearson correlations (r) and two-tailed significance levels (p) are reported separately for U.S. and Congolese samples.
